# Preparing a Community Hospital to Manage Work-related Exposures to Infectious Agents in BioSafety Level 3 and 4 Laboratories

**DOI:** 10.3201/eid1603.091485

**Published:** 2010-03

**Authors:** George F. Risi, Marshall E. Bloom, Nancy P. Hoe, Thomas Arminio, Paul Carlson, Tamara Powers, Heinz Feldmann, Deborah Wilson

**Affiliations:** Infectious Disease Specialists, PC, Missoula, Montana, USA (G.F. Risi); St. Patrick Hospital and Health Sciences Center, Missoula (T. Powers); National Institutes of Health, Bethesda, Maryland, USA (G.F. Risi, N.P. Hoe, T. Arminio, P. Carlson, D. Wilson); Rocky Mountain Laboratories, Hamilton, Montana, USA (M.E. Bloom, H. Feldmann)

**Keywords:** Viral hemorrhagic fevers, occupational exposure, curriculum, biosafety level, viruses, community hospital, perspective

## Abstract

Construction of new BioSafety Level (BSL) 3 and 4 laboratories has raised concerns regarding provision of care to exposed workers because of healthcare worker (HCW) unfamiliarity with precautions required. When the National Institutes of Health began construction of a new BSL-4 laboratory in Hamilton, Montana, USA, in 2005, they contracted with St. Patrick Hospital in Missoula, Montana, for care of those exposed. A care and isolation unit is described. We developed a training program for HCWs that emphasized the optimal use of barrier precautions and used pathogen-specific modules and simulations with mannequins and fluorescent liquids that represented infectious body fluids. The facility and training led to increased willingness among HCWs to care for patients with all types of communicable diseases. This model may be useful for other hospitals, whether they support a BSL-4 facility, are in the proximity of a BSL-3 facility, or are interested in upgrading their facilities to prepare for exotic and novel infectious diseases.

## Abstract 2

Training increased willingness of healthcare workers to care for patients with all types of communicable diseases.

Over the past decade, biomedical research performed on agents of viral hemorrhagic fevers (VHFs) has substantially increased. These agents are members of several virus groups, including filoviruses (Ebola virus, Marburg virus), Old World arenaviruses (Lassa virus, Lujo virus), New World arenaviruses (Machupo virus, Junin virus, Sabia virus, Guanarito virus, Chapare virus), flaviviruses (Omsk hemorrhagic fever virus, Kyasanur Forest disease virus), and bunyaviruses (Crimean–Congo hemorrhagic fever virus, Rift Valley fever virus) (*1*). Work with these agents is performed in specialized containment laboratories, operating at either BioSafety Level (BSL) 3 or BSL-4. BSL-3 denotes the potential for aerosol transmission to the laboratory worker. An agent that also is associated with high lethality and for which no available vaccine or specific treatment exists is studied at BSL-4 (*2*). Many VHF agents have a demonstrated potential for person-to-person transmission, including in nosocomial settings. A recent example of person-to-person transmission to hospital personnel occurred in September and October 2008 when Lujo virus was transmitted from the index patient to a paramedic, 2 nurses, and a member of the janitorial staff. Barrier precautions were not in place at the time of these events (*3*).

To provide safe work settings in which to study these pathogens, several BSL-4 laboratories are either in operation or under construction in the United States and abroad (Table 1) (T.G. Ksiazek, pers. comm.). Operation and management of these facilities are characterized by redundant engineering of safety features, strict administrative oversight, biosecurity measures, and extensive training (*2*,*4*), all designed to reduce the risk for exposure to persons working in this environment and prevent agents from being released into the community. Despite these safeguards, researchers in the United States and abroad have, on occasion, sustained occupational exposures to such agents, which rarely have resulted in overt illness and death (Table 2) (*5*–*11*). Because of the potential for person-to-person transmission of many VHF agents, rendering care to exposed or ill persons requires considerations beyond the scope of traditional hospital practices. Contact and/or airborne isolation guidelines may need to be added to standard isolation over the course of a patient’s hospitalization (*12*,*13*).

**Table 1:** BSL-4 laboratories planned or operational, 2009*

Location	Status
United States	
Centers for Disease Control and Prevention, Atlanta, GA, USA	A
Georgia State University Viral Immunology Center, Atlanta	A
Boston University National Emerging Infectious Disease Laboratories, Boston, MA, USA	NA
United States Army Medical Research Institute of Infectious Diseases, Fort Detrick, MD, USA	A
Department of Homeland Security National Biodefense Analysis and Countermeasures Center, Frederick, MD, USA	NA
National Institute of Allergy and Infectious Diseases	
Integrated Research Facility, Frederick	NA
Rocky Mountain Laboratories, Hamilton, MT, USA	A
Southwest Foundation for Biomedical Research, San Antonio, TX, USA	A
University of Texas Medical Branch, Galveston, TX, USA	
Robert E. Shope MD BSL-4 Laboratory	A
Galveston National Biocontainment Laboratory	A
Other countries	
Geelong, Victoria, Australia	A
Winnipeg, Ontario, Canada	A
Taiwan	NA
London and Salisbury, UK	A, A
Lyon, France	A
Libreville, Gabon	A
Hamburg, Marburg, Berlin, and Greifswald, Germany	A, A, A, NA
Pune, India	NA
Rome, Italy	A
Bilthoven, the Netherlands	A
Novosibirsk, Russia	A
Sandringham, South Africa	NA
Solna, Sweden	A
Geneva and Spiez, Switzerland	A, NA

*BSL-4, BioSafety Level 4; A, active; NA, nonactive.

**Table 2:** Infections caused by laboratory exposure to hemorrhagic fever viruses*

Virus	Incident
Ebola	Fingerstick while manipulating infected guinea pig tissue, 1977 (*5*); percutaneous exposure to blood from a Zaire Ebola virus–infected rodent, 2004 (*7*)
Marburg	3 laboratory acquired infections since the mid-1980s; 1 death occurred in Russia; no details available (*8*)
Crimean–Congo hemorrhagic fever	8 cases before 1980 compiled by SALS; no details available (*9*)
Lassa	1 case reported in 1970 with limited details provided (*10*)
Junin	21 cases before 1980 compiled by SALS; no details available (*9*)
Machupo	1 person exposed to aerosolized blood from a broken test tube (*11*)

*SALS, Subcommittee on Arbovirus Laboratory Safety.

On several occasions, persons naturally infected with a VHF agent have sought treatment at hospitals located in industrialized areas of the world (*14*–*21*). Often the correct diagnosis is not considered at the time of hospitalization, and only standard isolation is used until such time as the diagnosis is suspected or confirmed. Despite this limitation, nosocomial transmission of these agents is uncommon in adequately resourced hospitals (*16*,*18*,*20*,*21*). Notably, the medical care requirements for patients with a naturally acquired VHF illness are identical to those needed for laboratory-acquired infections with the same agents.

Because of the limited and unique settings in which BSL-4 research has historically taken place in the United States, hospitalization for occupational exposures to VHF agents has typically been a dedicated facility remote from a conventional hospital, e.g., the medical containment suite (the “slammer”) at the US Army Medical Research Institute of Infectious Diseases (USAMRIID), Frederick, Maryland, USA, or the biocontainment patient care unit at Emory University, Atlanta, Georgia, USA. The benefits of a remote facility include reducing the risk for nosocomial transmission, use of personnel who are already trained in managing a patient in containment, and control of public access (*22*). However, this approach has several serious drawbacks, including limited access to medical specialties and nursing staff, limited availability of medications and blood products, and limited access to specialized equipment such as ventilators and hemodialysis machines. In addition, increased psychological stress is experienced by patients confined to such a facility. Finally, given that the need to activate these facilities is extremely rare, the expense of building and maintaining a stand-alone unit poses a substantial limitation to this approach.

In addition to physical separation of the facility, medical and support staff at the USAMRIID facility work in positive pressure suits similar to those used in the laboratories themselves (*22*). Although the use of such suits provides protection to the caregiver, positive pressure suits are cumbersome, physically demanding to work in, and require substantial time for donning and doffing (dressing and undressing). Furthermore, venipuncture and other interventions in this unaccustomed and inconvenient setting pose a clear exposure risk to healthcare workers (HCWs). These factors are serious drawbacks when a HCW needs to render care to an acutely ill patient.

Documented clinical experience from several situations clearly indicates that nosocomial transmission can be prevented by implementing standard, contact, and airborne isolation procedures (*3*,*15*,*16*,*19**,*,*20*). Furthermore, all BSL-4 research programs stress the importance of recognizing and quickly reporting potential work-related exposures and illnesses to occupational medical and safety staff. Thus, healthcare staff will typically be informed about the specific agent and the nature of the exposure early in the incubation period. This will enable rapid evaluation and timely institution of appropriate isolation precautions.

Given all these considerations, what additional enhancements are really necessary for a hospital to safely care for patients while still enabling delivery of optimum medical care? Because of sensational misconceptions about VHF agents in popular media such as movies and the press, other serious issues are the willingness of HCWs to render care to such persons and how to determine what additional actions would increase the likelihood of their doing so. We offer a practical approach to dealing with these issues in the procedures followed by a patient isolation facility located in Missoula, Montana, USA, and its attendant training and educational components.

## Care and Isolation Unit

The Division of Intramural Research of the National Institute of Allergy and Infectious Diseases (NIAID) recently completed construction of an integrated research facility with BSL-4 research space at its Rocky Mountain Laboratories (RML) in Hamilton, Montana. As part of the project, NIAID contracted with St. Patrick Hospital and Health Sciences Center (SPH), a regional referral medical center located in Missoula, Montana, for provision and staffing of a patient isolation facility to support the RML BSL-4 research program. The facility, known as a care and isolation unit (CIU) (*23*) was designed to care for RML workers who had either known or had potential exposure to, or illness from, work-related diseases. The facility had to be located within 75 miles of RML, had to provide the full range of standard in-patient care, including intensive care, and had to meet the facility design guidelines of the National Institutes of Health, Division of Occupational Health and Safety (NIH DOHS) (*24*). Furthermore, the hospital had to supply the personnel to provide the full range of medical and nursing care and to be able to accept a patient within 8 hours (this would entail notification of key members of the hospital hierarchy, transferring patients if the rooms were currently occupied, securing adequate nursing and support staff, and carrying out systems checks to ensure that air handling systems and autoclaves were operational). In addition to the physical facility, a training program for critical care nurses, physicians, and other medical personnel was a major component of the contract.

To satisfy the NIH requirements for the CIU, the following elements were needed: 1) access control, i.e., the ability to restrict entrance into the CIU to authorized persons only; 2) three separate stand-alone rooms, each with a bathroom and shower, separate air handling, and an anteroom separating the patient room from the hallway; 3) directional air flow from the hallway into the anteroom and from the anteroom into the patient room; 4) a dedicated exhaust system providing >12 air exchanges per hour to the patient rooms (including >2 outside air changes per hour); 5) passage of exhaust through a HEPA filter to the building exterior >8 feet above the rooftop and well removed from air intake ducts; 6) room surfaces constructed of seamless materials amenable to topical disinfection; 7) the capability for the full range of intensive care unit (ICU) monitoring and support, including the ability to perform limited surgery, hemodialysis or peritoneal dialysis, Swan-Ganz catheter placement, and hemodynamic monitoring; and 8) a separate autoclave within the CIU for sterilizing all items that come out of a patient room.

SPH was selected to provide these services and facilities. SPH is a not-for-profit medical center under the sponsorship of the Sisters of Providence. It has 195 acute care beds, and >10,000 patient admissions per year. The full range of standard specialty medical care is available within the hospital, including 24 hour, 7 day/week availability of specialists in critical care, infectious disease, and all surgical subspecialties.

SPH retrofitted 3 adjacent rooms within the existing medical ICU (MICU) to create the CIU. A set of doors was installed to control access to the CIU from the MICU, and these would remain open when the CIU was not in use (Figure). A separate fully equipped nursing station was constructed, with closed circuit television monitoring for each of the 3 rooms. After construction, the CIU was inspected and approved by officials from NIH DOHS. Under normal circumstances, the CIU operates either as 3 conventional MICU rooms or as isolation rooms for patients with community-acquired illnesses for which isolation of airborne pathogens is needed. If a patient from RML should require admission, any current occupants would be transferred, and access would be limited by closing off that section of the MICU.

**Figure:**
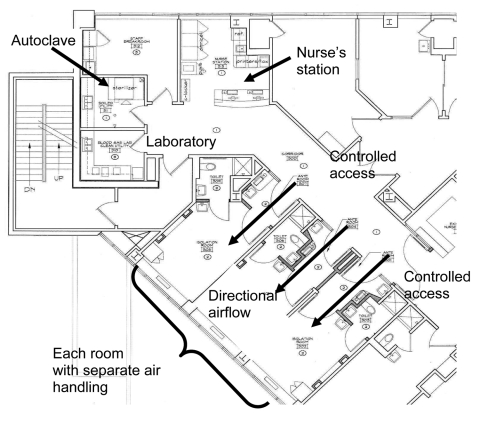
Floor plan of the Care and Isolation Unit, St. Patrick Hospital and Health Sciences Center, Missoula, MT, USA.

In addition to the physical aspects of the CIU, several other elements were developed. Specific policies and procedures were written that deal with all aspects from admission to discharge, including unique aspects such as clean up of infected bodily spills, donning and doffing of personal protective equipment (PPE), and use of the autoclave. Support of hospital administration, physicians, nurses, and support personnel was critical. This backing was enlisted primarily by mounting an educational campaign that stressed the true risk for nosocomial transmission of these agents, as well as the recognition that the increased resources that would be provided to the hospital could greatly enhance capacity for handling community-acquired infections.

One feature dealt with preparing the hospital staff to care for such exposed persons. To accomplish this feature, we developed a detailed curriculum, which can be presented during a 1-day training workshop. This workshop includes didactic information, patient care scenarios discussed in group settings, and hands-on training. Simulation of various patient care activities (hand hygiene, donning and doffing of PPE, cleanup of body fluids, and rendering ICU level care to a patient) is conducted by using programmable mannequins and either tonic water or Glo Germ (Glo Germ, Moab, UT, USA), both of which fluoresce under ultraviolet light, to simulate infectious body fluids. Continuing education credits are granted for participation. Competence is maintained with quarterly demonstration of proper technique, review of CIU-specific policies and procedures, and required utilization of a series of online problem-oriented patient care scenarios. Training videos have been developed that demonstrate proper technique for spill cleanup, donning and doffing of PPE, processing of patient specimens, and processing of biohazardous waste, including use of the autoclave. Finally, detailed educational modules have been developed for each of the BSL-4 pathogens. These modules are designed to provide a nurse, emergency medical technician, or critical care physician with critical information that is quickly accessible as well as an extensive discussion of all aspects of the agent. The modules are in a standard format with extensive references and websites for further reading. All of this information is available for review any time both in hard copy as well as on the hospital’s intranet site in the form of slide presentations, videos, or PDF files. The SPH staff has been generous in supplying feedback on the training and has been instrumental in refining the curriculum. Acquisition of knowledge has been documented with the use of pretesting and posttesting. After completion of the training, SPH staff members expressed increased confidence in caring for patients with all types of communicable infectious diseases, including VHFs.

To maintain readiness, a series of drills and exercises have been performed and will continue, in collaboration with RML and local emergency medical services providers. These readiness exercises have encompassed all aspects of care from arrival to the hospital through discharge.

## Discussion

Engineering and administrative controls as well as PPE and standard operating procedures that are in place in modern BSL-4 laboratories have been associated with a greatly reduced incidence of occupational exposures to infectious agents (*23*,*25*). However, exposures, now primarily by the percutaneous route, still occur. USAMRIID recently published a review of potential laboratory exposures to agents of bioterrorism at their facility during 1989–2002 (*26*). During that time, 12 evaluations were made for potential exposures to filoviruses (Ebola virus or Marburg virus), 3 to arenaviruses, and 4 to Crimean–Congo hemorrhagic fever virus. Although none of these incidents was deemed a high enough risk to warrant isolation of the exposed persons, 2 laboratory workers were given investigational antiviral agents. One exposure at USAMRIID in 2004 resulted in isolation when a scientist received a puncture injury through a gloved hand while manipulating a mouse that had been experimentally exposed to Ebola virus (*22*). Fortunately, none of these situations resulted in infection. However, workers have been infected by agents of VHF from laboratory accidents elsewhere (Table 2).

Nosocomial transmission of VHF is infrequently described outside of resource-poor settings. With rare exception, such events have occurred because of the lack of recognition that the index patient had such an infection (*3*,*18*). The Centers for Disease Control and Prevention (CDC) has published guidelines for management of patients infected with viral hemorrhagic fevers in the conventional hospital setting (*12*,*13*). Notably, medical care has been safely rendered by using conventional barrier precautions alone to persons infected with VHF viruses, including Ebola virus (*5*,*18*), Marburg virus (*19**,**20*), Lassa fever virus (*27*), Machupo virus (*11*), Sabia virus (*28*), and Crimean–Congo hemorrhagic fever virus (*21*).

Nevertheless, even well-trained HCWs may make mistakes due to anxiety, fatigue, or other stressors, so additional facility enhancements that augment safety are desirable when dealing with potentially lethal infectious diseases. Furthermore, the recognition of a patient with an exotic or unfamiliar contagious disease may engender trepidation among the medical community as well as the public. Such concerns have at times resulted in reluctance on the part of HCWs to care for persons infected with such agents as monkeypox virus (*29*), *Yersinia pestis* (plague) (*30*), and others. When a sample of 1,000 physicians were surveyed (526 responses), 80% indicated a willingness to care for patients in the event of an outbreak of an unknown but potentially deadly illness, but only 21% felt adequately prepared to do so (*31*). Reluctance is often out of proportion to the true risks and results from concerns for personal and family member safety. These concerns are likely to be reduced if the HCW perceives that the facility has taken additional precautions and instituted additional training.

To maximize safety as well as to address provider concerns of HCWs and other staff, we have developed the CIU and our accompanying training program. Our pragmatic and practical approach provides a well-designed facility that enhances safety not only for the care of a patient infected with a laboratory-acquired VHF virus infection, but also for serious transmissible community-acquired disease or for exotic diseases contracted while traveling.

As international tourism and work assignments continue to expand, the importation of exotic diseases is almost certain to increase and to appear in unexpected locations. Recent instances of infection have occurred with Marburg virus in Colorado (*19*) and the Netherlands (*20*); with Lassa fever virus in New Jersey (*15*), the United Kingdom (*16*), and Germany 6 (*16*); with *Y. pestis* (*32*) in New York, New York; and with (initially thought) extensively drug-resistant *Mycobacterium tuberculosis* in Atlanta, Georgia (*33*). Finally, in the United States, 1,356 BSL-3 laboratories are registered with either CDC or the US Department of Agriculture select agent programs (*34*). For these reasons, relatively low-cost facilities ($624,000.00 for design and construction of our unit) like the CIU may become more critical. Furthermore, training programs, similar to the one we have implemented, with emphasis on such practical infection control issues as the proper use of PPE, hand hygiene, and proper spill cleanup, has broad application. Other communities might consider the benefits of our approach, whether or not infectious disease research laboratories are constructed in their area.
